# Prevalence and genetics of cefepime resistance among the AmpC-producing organisms *Citrobacter freundii* complex, *Enterobacter cloacae* complex, and *Klebsiella aerogenes*

**DOI:** 10.1128/aac.01661-25

**Published:** 2026-03-27

**Authors:** Sarah M. Schrader, Zachary Pearson, Samantha Taffner, Sanjat Kanjilal, Nicole D. Pecora

**Affiliations:** 1 Department of Pathology, Brigham and Women’s Hospital 550233, Boston, Massachusetts, USA; 2 Department of Pathology, Massachusetts General Hospital 272105, Boston, Massachusetts, USA; 3 Mass General Brigham 1813, Boston, Massachusetts, USA; 4 Department of Population Medicine, Harvard Medical School and Harvard Pilgrim Health Care Institute, Boston, Massachusetts, USA; 5 Division of Infectious Diseases, Department of Medicine, Brigham and Women's Hospital 370908, Boston, Massachusetts, USA; 6 Department of Medical Microbiology and Infection Prevention, Amsterdam University Medical Center 522567, Amsterdam, the Netherlands; Entasis, Big Bay, Michigan, USA

**Keywords:** BCID2, molecular diagnostics, *Klebsiella aerogenes*, *Citrobacter freundii*, *Enterobacter cloacae*, AmpC, cefepime, ESBL

## Abstract

Cefepime is generally recommended for treatment of clinically significant AmpC producers, including *Citrobacter freundii* complex (*Cfre*), *Enterobacter cloacae* complex (*Eclo*), and *Klebsiella aerogenes* (*Kaer*). Clinicians increasingly use the results of multiplex PCR panels to make early treatment decisions, with cefepime often chosen when an AmpC producer is detected without a *bla*_CTX-M_ extended-spectrum beta-lactamase (ESBL) or carbapenemase. To understand the safety of this approach, we evaluated the prevalence of cefepime resistance among 4,687 *Cfre*; 9,878 *Eclo*; and 4,800 *Kaer* clinical isolates collected in our hospital network in New England, USA, from 2015 to 2024. We additionally reviewed Blood Culture Identification 2 (BCID2) (bioMérieux) results for 136 blood cultures and the sequenced genomes of 184 isolates (14 cefepime-susceptible dose-dependent [SDD], 34 cefepime-resistant). Cefepime-SDD and cefepime-resistant isolates made up 0.4% (18/4,687) and 1.3% (59/4,687) of *Cfre*, 2.0% (194/9,879) and 3.0% (299/9,879) of *Eclo*, and 0.3% (16/4,800) and 0.6% (28/4,800) of *Kaer*. Of 117 *Eclo* and *Kaer* identified in blood cultures on the BCID2, 4.3% (5/117) were cefepime-SDD or cefepime-resistant and negative for an ESBL or carbapenemase target. Among sequenced cefepime-SDD isolates, 21% (3/14) carried an ESBL. Cefepime-resistant isolates carried diverse beta-lactamase genes, including ESBL genes, carbapenemase genes, and plasmid-borne *ampC*. While reduced cefepime susceptibility was generally associated with carriage of more beta-lactamase genes, 57% of cefepime-SDD and 35% of cefepime-resistant isolates encoded only chromosomal *ampC*. Our results highlight variable cefepime susceptibility rates for AmpC producers across clinical settings and demonstrate diverse mechanisms underlying reduced cefepime susceptibility.

## INTRODUCTION

Organisms bearing chromosomal AmpC beta-lactamases present significant treatment challenges. They are intrinsically resistant to beta-lactams that are both potent inducers of and good substrates for AmpC, including aminopenicillins, first-generation cephalosporins, and cephamycins (1), and frequently exhibit inducible resistance to beta-lactams that are weak inducers of but good substrates for AmpC, including piperacillin-tazobactam, ceftriaxone (CRO), ceftazidime, and aztreonam (1). Due to the inducible nature of AmpC expression, isolates that initially test susceptible to these antibiotics rapidly become resistant upon exposure, presenting both diagnostic and treatment dilemmas (2).

*Citrobacter freundii* complex (*Cfre*), *Enterobacter cloacae* complex (*Eclo*), and *Klebsiella aerogenes* (*Kaer*) pose the highest risk of clinically significant AmpC production (1). Because of this risk, the Infectious Diseases Society of America (IDSA) recommends cefepime (FEP)—a weak inducer of and poor substrate for AmpC (1)—for the treatment of infections caused by these organisms. For isolates that are FEP-resistant (R), carbapenems are typically chosen. While high-dose FEP can be considered for the treatment of isolates with a FEP minimum inhibitory concentration (MIC) in the susceptible dose-dependent (SDD) range, FEP is considered inferior to carbapenems for FEP-SDD isolates that co-produce extended-spectrum beta-lactamases (ESBLs) such as *bla*_CTX-M_ (1). Reduced FEP susceptibility often reflects ESBL and/or carbapenemase carriage, but constitutive derepression of AmpC and loss of outer membrane porins can also raise FEP MICs (1–3).

To support optimal clinical treatment decisions, clinical microbiology laboratories are increasingly implementing multiplex PCR panels for use directly on patient specimens or on positive blood cultures. Many such panels can identify Enterobacterales species, including AmpC producers, along with select resistance determinants such as *bla*_CTX-M_ and the major carbapenemases *bla*_KPC_, *bla*_NDM_, *bla*_IMP_, *bla*_VIM_, and *bla*_OXA-48-like_. While these tools are currently limited to select specimen types, they influence the selection of empiric therapy in a substantial number of patients. In our network, FEP is often chosen when no resistance determinants are detected. However, this approach has a potential pitfall: given the diversity of mechanisms underlying FEP-SDD and FEP-R phenotypes in AmpC producers (1–3), clinicians risk choosing inappropriately narrow therapy until phenotypic susceptibility results arrive, typically 2 days later. The extent to which gene targets or mutations not represented on commercially available panels appear in clinical isolates that are eventually found to have elevated FEP MICs and the distribution of underlying molecular determinants for this phenotype are currently unknown.

We sought to assess the safety of FEP for the empiric treatment of AmpC-producing organisms in the era of rapid molecular diagnostics by estimating the prevalence of FEP-SDD and -R phenotypes in our region and defining the underlying molecular determinants in i) a subset of bloodstream isolates that were analyzed with the BIOFIRE Blood Culture Identification 2 (BCID2) panel (bioMérieux) and ii) a separate subset that underwent whole-genome sequencing (WGS).

## RESULTS

### Prevalence of FEP-SDD and FEP-R isolates among *Cfre*, *Eclo*, and *Kaer* clinical isolates

**Fig 1:**
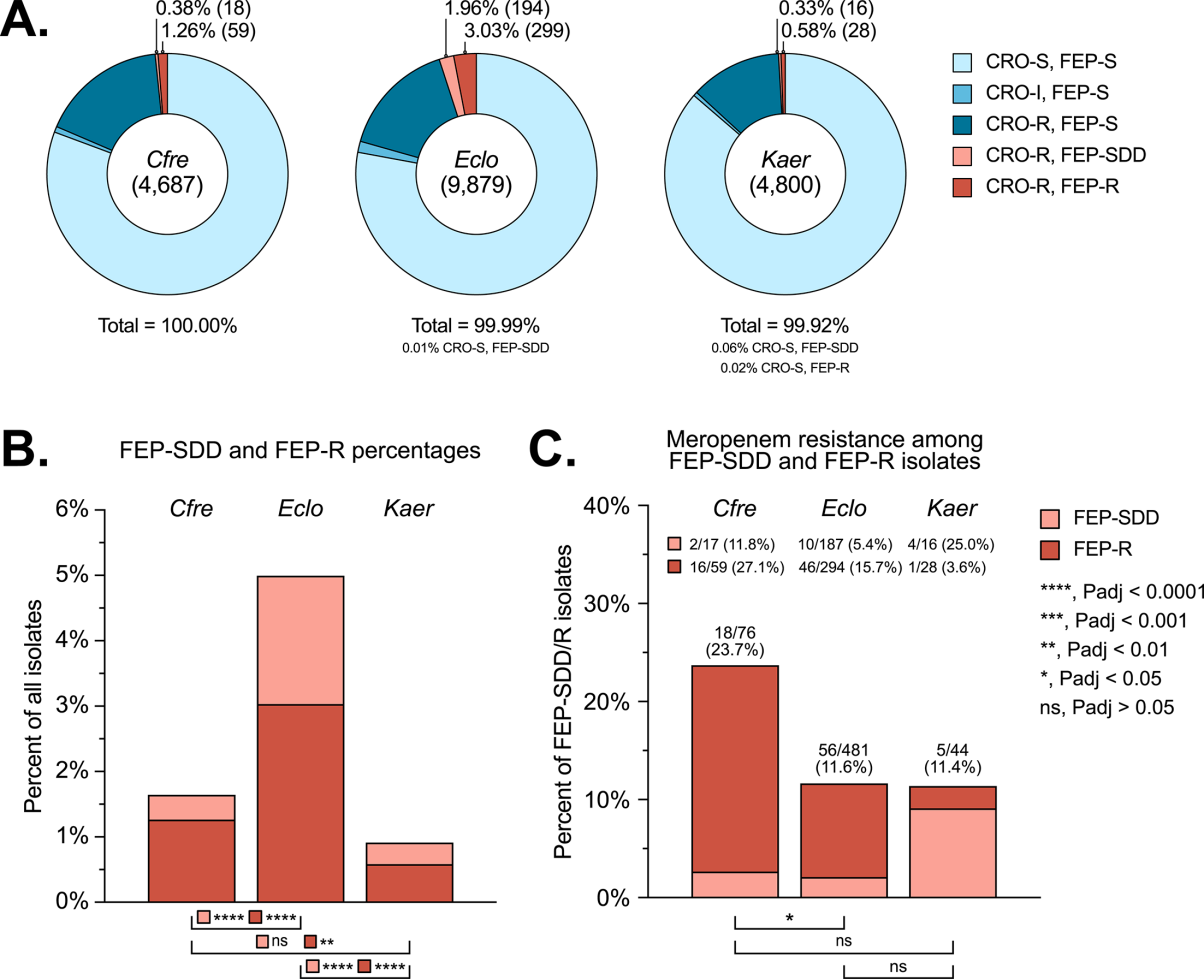
Prevalence of ceftriaxone-resistant (CRO-R), cefepime susceptible-dose-dependent (FEP-SDD) and CRO-R, cefepime-resistant (FEP-R) phenotypes among *Cfre*, *Eclo*, and *Kaer* clinical isolates. (**A**) CRO and FEP susceptibility profiles for each organism group. S, susceptible. I, intermediate. SDD, susceptible dose-dependent. R, resistant. The total number of isolates is indicated in parenthesis in the center of each chart. CRO-R, FEP-SDD, and CRO-R, FEP-R categories are labeled with the percentage and number of isolates (in parenthesis). Numbers for other categories are available in Table S1. (**B**) Comparison of the percentages of total isolates that exhibited the CRO-R, FEP-SDD (light red) or CRO-R, FEP-R (dark red) phenotype across the three organism groups. (**C**) Percentages of FEP-SDD (light red) and FEP-R (dark red) isolates that were resistant to meropenem across the three organism groups. The numbers directly above each bar represent the total number of FEP-SDD and FEP-R isolates that were meropenem-resistant over the total number of FEP-SDD and FEP-R isolates with the corresponding percentage in parenthesis. The numbers under the organism abbreviations show these totals broken down by FEP phenotype: the numbers in line with the light red box correspond to the total number of FEP-SDD isolates that were meropenem-resistant over the total number of FEP-SDD isolates, and the numbers in line with the dark red box correspond to the total number of FEP-R isolates that were meropenem-resistant over the total number of FEP-R isolates (corresponding percentages in parenthesis). For B and C, percentages were compared using the chi-square test. *P*-values were adjusted for multiple comparisons using the Bonferroni correction (Padj; based on six tests for B and three tests for C).

Our study set comprised 19,366 clinical isolates: 4,687 *Cfre*, 9,879 *Eclo*, and 4,800 *Kaer*. Most (80.7% of *Cfre*, 77.9% of *Eclo*, and 86.2% of *Kaer*) were susceptible to both CRO and FEP (Fig. 1A and Table 1; Table S1). Isolates resistant to CRO but susceptible to FEP were next most common (16.9% of *Cfre*, 15.6% of *Eclo*, and 12.2% of *Kaer*). The prevalence of both FEP-SDD and FEP-R isolates was highest for *Eclo* (2.0% FEP-SDD vs 0.4% for *Cfre*, *P* < 0.001, and 0.3% for *Kaer*, *P* < 0.001; 3.0% FEP-R vs 1.3% for *Cfre*, *P* < 0.001, and 0.6% for *Kaer*, *P* < 0.001) (Fig. 1B). Among FEP-SDD and FEP-R isolates that were tested for susceptibility to meropenem (MEM), 23.7% of *Cfre* isolates, 11.6% of *Eclo* isolates, and 11.4% of *Kaer* isolates were resistant (Fig. 1C, Table 1), classifying them as carbapenem-resistant Enterobacterales (CRE).

**TABLE 1:** Clinical Laboratory Standards Institute (CLSI) ceftriaxone (CRO), cefepime (FEP), and meropenem (MEM) breakpoints^*a*^

Interpretive category	CRO		FEP		MEM	
	MIC (µg/mL)	Zone diameter (mm)	MIC (µg/mL)	Zone diameter (mm)	MIC (µg/mL)	Zone diameter (mm)
S	≤1	≥23	≤2	≥25	≤1	≥23
SDD	N/A	N/A	4–8	19–24	N/A	N/A
I	2	20–22	N/A	N/A	2	20–22
R	≥4	≤19	≥16	≤18	≥4	≤19

^*a*^ From Table 2A-1 of the 35th edition of the CLSI M100 Performance Standards for Antimicrobial Susceptibility Testing. S, susceptible. SDD, susceptible dose-dependent. I, intermediate. R, resistant. MIC, minimum inhibitory concentration. N/A, not applicable.

**TABLE 2:** Characteristics of whole-genome-sequenced ceftriaxone-resistant, cefepime-susceptible dose-dependent isolates^*a*^

Species	ST	Year	Setting	Specimen type	CRO MIC	FEP MIC	MEM MIC	Beta-lactamases
*C. freundii*	64	2022	IP	Joint	≥64 (R)	8 (SDD)	≤0.25 (S)	*bla*_CMY_ (C) *bla*_TEM-1_ (A) *bla*_LAP-2_ (A)
*C. freundii*	232	2022	IP	Respiratory tract	8 (R)	4 (SDD)	≤0.25 (S)	*bla*_CMY_ (C)
*E. chengduensis*	598	2021	IP	Rectal	≥64 (R)	8 (SDD)	1 (S)	*bla*_ACT-48_ (C)
*E. cloacae*	Undetermined	2022	IP	Respiratory tract	≥64 (R)	4 (SDD)	≤0.25 (S)	*bla*_ACT_ (C)
*E. cloacae*	114	2024	OP	Skin/tissue	16 (R)	4 (SDD)	≤0.25 (S)	*bla*_ACT-16_ (C) *bla*_TEM-1_ (A)
*E. hormaechei*	45	2022	OP	Skin/tissue	≥64 (R)	4 (SDD)	≤0.25 (S)	*bla*_ACT-17_ (C) *bla*_TEM-1_ (A)
*E. hormaechei*	106	2023	ED	Urine	≥64 (R)	4 (SDD)	1 (S)	*bla*_ACT-56_ (C) *bla*_CTX-M-15_ (A, ES) *bla*_TEM-1_ (A) *bla*_OXA-1_ (D)
*E. hormaechei*	116	2022	OP	Urine	≥64 (R)	8 (SDD)	≤0.25 (S)	*bla*_ACT-17_ (C)
*E. hormaechei*	116	2023	OP	Urine	≥64 (R)	4 (SDD)	0.5 (S)	*bla*_ACT-17_ (C)
*E. hormaechei*	171	2023	IP	Blood	≥64 (R)	4 (SDD)	≤0.25 (S)	*bla*_ACT_ (C) *bla*_SHV-12_ (A, ES)
*E. hormaechei*	604/662	2022	IP	Respiratory tract	≥64 (R)	4 (SDD)	≤0.25 (S)	*bla*_ACT-17_ (C)
*E. hormaechei*	974	2024	IP	Skin/tissue	≥64 (R)	4 (SDD)	≤0.25 (S)	*bla*_ACT-25_ (C)
*E. hormaechei*	974	2023	IP	Respiratory tract	≥64 (R)	4 (SDD)	≤0.25 (S)	*bla*_ACT_ (C) *bla*_CTX-M-15_ (A, ES) *bla*_TEM-1_ (A) *bla*_OXA-1_ (D)
*E. hormaechei*	1,073	2022	IP	Urine	≥64 (R)	8 (SDD)	≤0.25 (S)	*bla*_ACT-69_ (C)

^*a*^ ST, sequence type. Setting, clinical setting in which the specimen was collected. IP, inpatient. OP, outpatient. ED, emergency department. CRO, ceftriaxone. FEP, cefepime. MEM, meropenem. MIC, minimum inhibitory concentration. MICs are in units of µg/mL. The interpretive category according to the breakpoints in the 35^th^ edition of the CLSI M100 is indicated in the parenthesis after each value. S, susceptible. SDD, susceptible dose-dependent. R, resistant. Beta-lactamase genes detected via annotation of whole-genome sequences are listed with the Ambler class (A–D) in parenthesis. Presumed extended-spectrum beta-lactamase genes are annotated as “ES,” and presumed carbapenemase genes are annotated as “CP”.

### Prevalence of FEP-SDD and FEP-R isolates by patient location and specimen type

**Fig 2:**
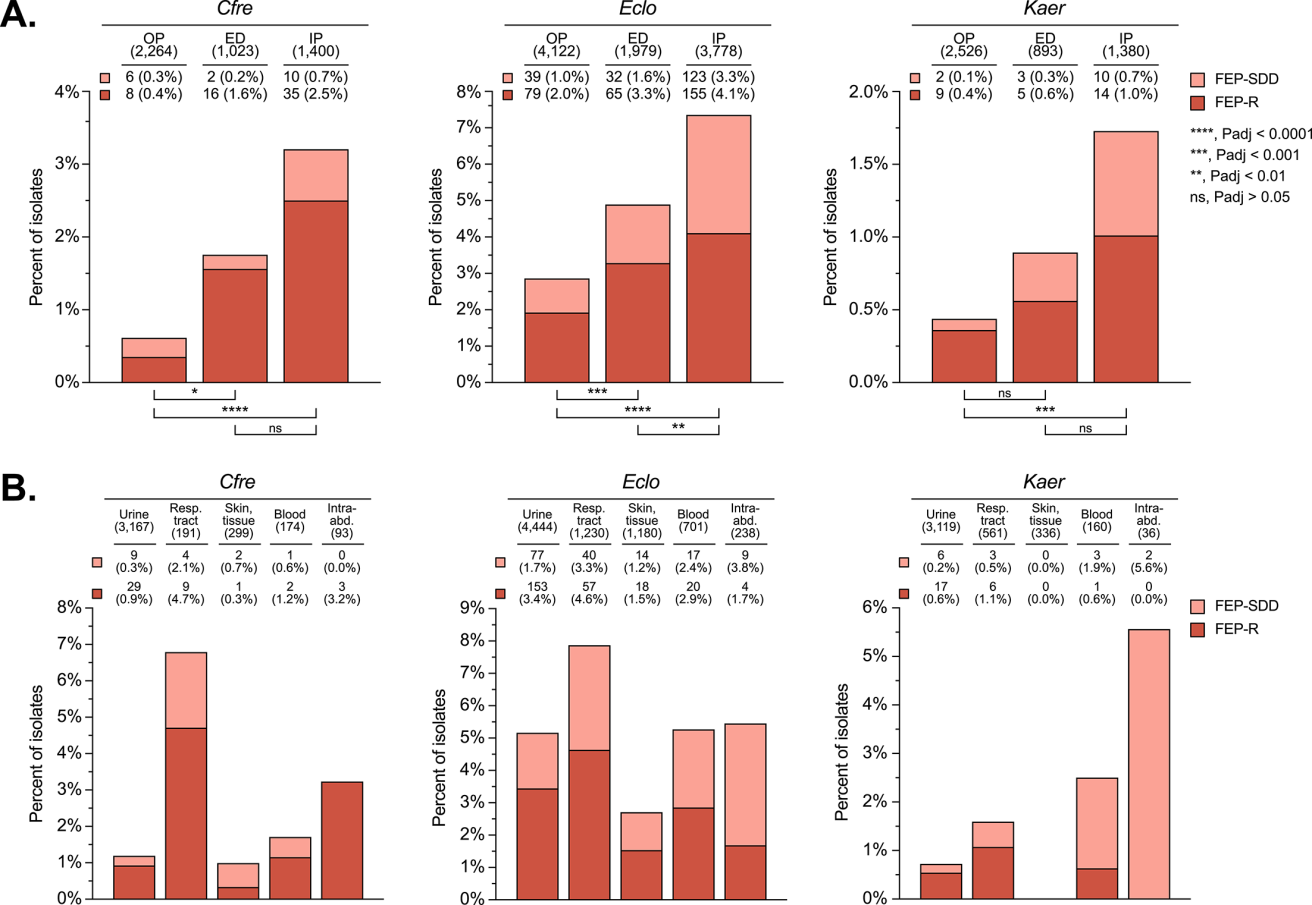
Prevalence of ceftriaxone-resistant (CRO-R), cefepime susceptible-dose-dependent (FEP-SDD), and CRO-R, cefepime-resistant (FEP-R) isolates in *Cfre*, *Eclo*, and *Kaer* by patient location and specimen type. For both panels, the number of isolates in each category is indicated in parenthesis under the category name at the top of each graph. The numbers of FEP-SDD (in line with the light red box) and FEP-R (in line with the dark red box) isolates represented by each bar are indicated with the percentages in parenthesis. (**A**) CRO-R, FEP-SDD, and CRO-R, FEP-R prevalence stratified by patient location at the time of specimen collection. OP, outpatient. ED, emergency department. IP, inpatient. The total percentages represented by each bar (i.e., CRO-R, FEP-SDD, plus CRO R, FEP-R) were compared using the chi-square test. *P*-values were adjusted for multiple comparisons using the Bonferroni correction (based on nine tests). One *Kaer* isolate was omitted due to an unknown patient location at the time of specimen collection. (**B**) CRO-R, FEP-SDD, and CRO-R, FEP-R prevalence stratified by specimen type. The five most common specimen types were included. Resp. tract, respiratory tract. Intra-abd., intra-abdominal.

The prevalence of FEP-SDD and FEP-R isolates was highest among isolates collected in inpatient settings (Fig. 2A). For *Cfre*, *Eclo*, and *Kaer*, 3.2% (45/1,400), 7.4% (278/3,778), and 1.7% (24/1,380) of isolates from inpatients were FEP-SDD/R compared to 1.8% (18/1,023, *P* > 0.05), 4.9% (97/1,979, *P* < 0.01), and 0.9% (8/893, *P* > 0.05) of isolates from emergency department patients and 0.6% (14/2,264, *P* < 0.0001), 2.9% (118/4,122, *P* < 0.0001), and 0.4% (11/2,526, *P* < 0.001) of isolates from outpatients, respectively. Among specimen types (Fig. 2B), respiratory tract isolates had the highest prevalence of FEP-SDD and FEP-R for *Cfre* (6.8%, 13/191) and *Eclo* (7.9%, 97/1,230). For bloodstream isolates, 4.3% (44/1,035) across all organism groups were FEP-SDD or FEP-R, with the highest prevalence for *Eclo* (5.3%, 37/701).

**Fig 3:**
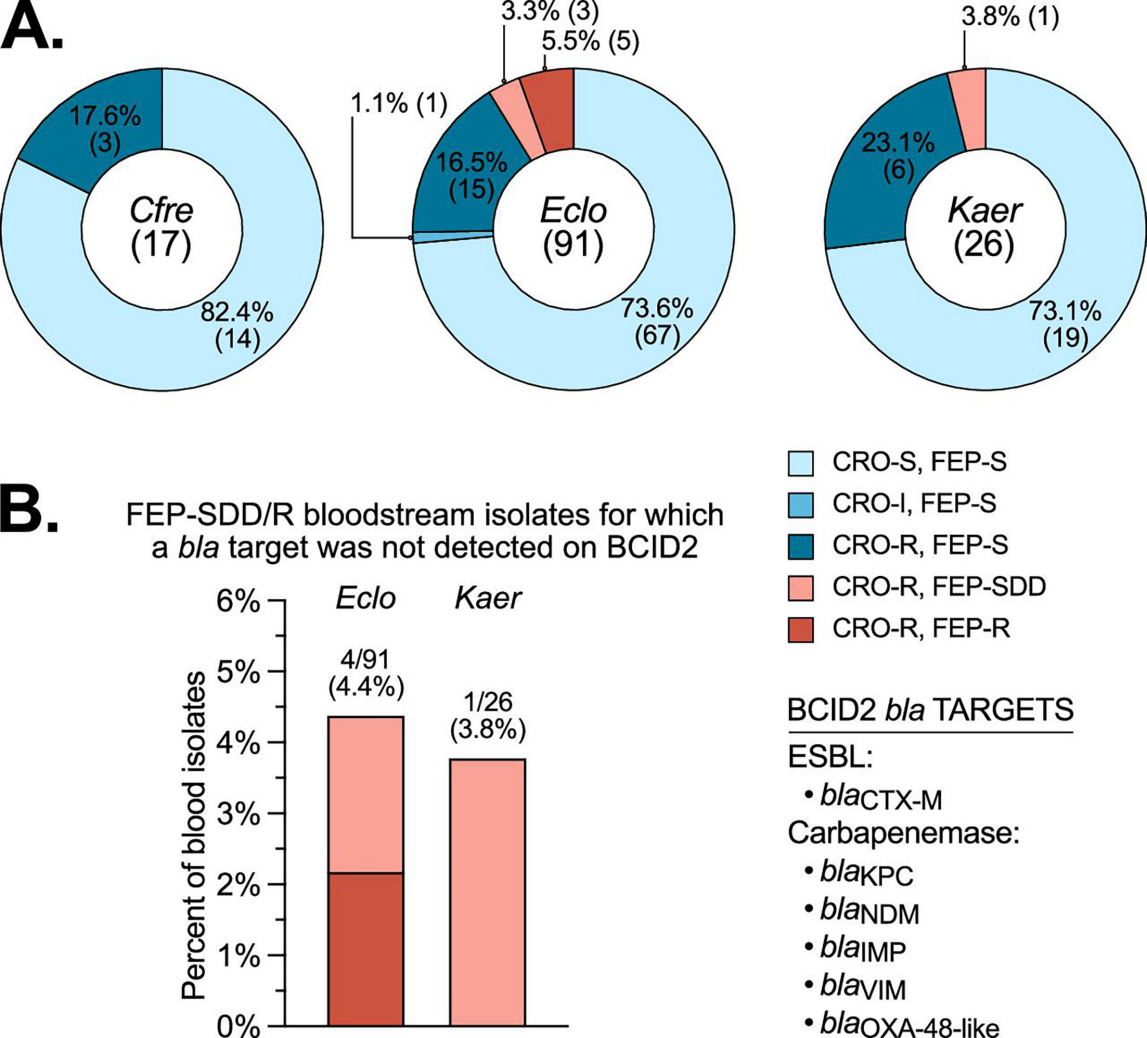
Cefepime (FEP) susceptibility of *Cfre*, *Eclo*, and *Kaer* bloodstream isolates detected by the BIOFIRE Blood Culture Identification 2 (BCID2) rapid molecular panel. (**A**) Ceftriaxone (CRO) and FEP susceptibility profiles for each organism. Organisms were detected in positive blood culture bottles to the order (*Cfre*) or species/complex level (*Eclo* and *Kaer*) by BCID2 prior to culture for formal identification and phenotypic antimicrobial susceptibility testing. S, susceptible. I, intermediate. SDD, susceptible dose-dependent. R, resistant. The total number of isolates is indicated in parenthesis in the center of each chart. Each susceptibility category is labeled with the percentage and number of isolates (in parenthesis). (**B**) Percentage of FEP-SDD/R bloodstream isolates for which a beta-lactamase (*bla*) target was not detected on BCID2. BCID2 *bla* targets are listed to the right of the graph. The total number of isolates (FEP-SDD plus FEP R) represented by each bar is indicated above the bar, with the percentage in parenthesis.

Among *Eclo* bloodstream isolates run on the BCID2 multiplex PCR panel, 3.3% (3/91 from 87 patients) were FEP-SDD and 5.5% (5/91) were FEP-R (Fig. 3A). Of these, 2/3 (66.7%) FEP-SDD and 2/5 (40%) FEP-R isolates had no beta-lactamase target detected (Fig. 3B); *bla*_CTX-M_ was detected in the other four isolates. Among *Kaer* bloodstream isolates run on the BCID2 panel, 3.8% (1/26 from 24 patients) were FEP-SDD (Fig. 3A), with no beta-lactamase target detected (Fig. 3B). Seventeen blood cultures grew *Cfre* following detection of the Enterobacterales order-level target on the BCID2 with no complex- or species-level target; all isolates were FEP-S (Fig. 3A). All FEP-SDD/R isolates across all organisms were susceptible to MEM.

### Beta-lactamase carriage in FEP-SDD and FEP-R isolates

To understand the molecular basis of FEP-SDD and FEP-R phenotypes, we examined beta-lactamase carriage in the genomes of 44 *Cfre* (37 FEP-S, 2 FEP-SDD, and 5 FEP-R), 101 *Eclo* (67 FEP-S, 12 FEP-SDD, and 22 FEP-R), and 39 *Kaer* (32 FEP-S and 7 FEP-R) (Tables 2 and 3; Table S2). All isolates were CRO-R and had chromosomal *ampC* with a variety of alleles represented. For *Cfre*, 95.5% (42/44) had *bla*_CMY_ *ampC* genes, while the remaining 5.5% (2/44) had *bla*_CFE_ *ampC* genes. No alleles were specific to FEP-SDD/R isolates. For *Eclo*, 92.1% (93/101) had a *bla*_ACT_ *ampC* gene. The remaining 8, all FEP-S, had either a *bla*_MIR_ (5.0%, 5/101) or *bla*_CMH_ (3.0%, 3/101) *ampC* gene. While most FEP-SDD and FEP-R isolates with identified *bla*_ACT_ alleles carried alleles also found in FEP-S isolates, *bla*_ACT-48_ was present only in FEP-SDD isolates, while *bla*_ACT-24_ and *bla*_ACT-41_ were present only in FEP-R isolates (Fig. S1). *Kaer* isolates had a distinct *ampC* gene type from those of the other AmpC-producing organisms; allele-level identifications were not available.

**TABLE 3:** Characteristics of whole-genome-sequenced ceftriaxone-resistant, cefepime-resistant isolates^*a*^

Species	ST	Year	Setting	Specimen type	CRO MIC	FEP MIC	MEM MIC	Beta-lactamases
*C. cronae*	Undetermined	2023	IP	Respiratory tract	≥64 (R)	16 (R)	2 (I)	*bla*_CMY_ (C)
*C. freundii*	8	2022	IP	Blood	≥64 (R)	≥64 (R)	≥8 (R)	*bla*_CMY_ (C) *bla*_VEB-9_ (A, ES) *bla*_TEM_ (A) *bla*_KPC-2_ (A, CP) *bla*_OXA-9_ (D)
*C. freundii*	22	2024	IP	Rectal	≥64 (R)	≥64 (R)	≥16 (R)	*bla*_CMY-48_ (C) *bla*_SHV-12_ (A, ES) *bla*_TEM-1_ (A) *bla*_KPC-4_ (A, CP) *bla*_OXA-1_ (D)
*C. freundii*	64	2023	IP	Rectal	≥64 (R)	32 (R)	≥16 (R)	*bla*_CMY-152_ (C) *bla*_FOX-5_ (C, PAC) *bla*_TEM-1_ (A) *bla*_KPC-3_ (A, CP) *bla*_NDM-1_ (B, CP) *bla*_OXA-9_ (D)
*C. portucalensis*	155	2023	IP	Urine	≥64 (R)	≥ 64 (R)	≥16 (R)	*bla*_CMY_ (C) *bla*_DHA-1_ (C, PAC) *bla*_SHV-12_ (A, ES) *bla*_NDM-5_ (B, CP)
*E. absuriae*	252	2023	IP	Urine	≥64 (R)	32 (R)	≥16 (R)	*bla*_ACT-3_ (C) *bla*_SHV-7_ (A, ES) *bla*_KPC-4_ (A, CP) *bla*_OXA-1_ (D)
*E. bugandensis*	Undetermined	2022	IP	Respiratory tract	≥64 (R)	32 (R)	≤0.25 (S)	*bla*_ACT_ (C)
*E. cloacae*	114	2024	OP	Skin/tissue	≥64 (R)	≥64 (R)	≤0.25 (S)	*bla*_ACT-16_ (C) *bla*_SHV-1_ (A) *bla*_TEM-1_ (A)
*E. cloacae*	231	2022	OP	Urine	≥64 (R)	≥64 (R)	≥16 (R)	*bla*_ACT-25_ (C) *bla*_TEM-1_ (A) *bla*_NDM-1_ (B, CP)
*E. cloacae*	231	2021	ED	Urine	≥64 (R)	≥64 (R)	≥16 (R)	*bla*_ACT-25_ (C) *bla*_CTX-M-15_ (A, ES) *bla*_SHV-12_ (A, ES) *bla*_TEM-1_ (A) *bla*_NDM-1_ (B, CP) *bla*_OXA_ (D) *bla*_OXA-1_ (D)
*E. hormaechei*	50	2023	OP	Urine	≥64 (R)	16 (R)	2 (I)	*bla*_ACT-15_ (C)
*E. hormaechei*	50	2023	IP	Blood	≥64 (R)	≥64 (R)	1 (S)	*bla*_ACT-15_ (C) *bla*_CTX-M-15_ (A, ES) *bla*_OXA-1_ (D)
*E. hormaechei*	78	2023	OP	Skin/tissue	≥64 (R)	≥64 (R)	≤0.25 (S)	*bla*_ACT-24_ (C) *bla*_CTX-M-15_ (A, ES) *bla*_TEM-1_ (A) *bla*_OXA-1_ (D)
*E. hormaechei*	78	2024	IP	Rectal	≥64 (R)	≥64 (R)	14 mm (R)	*bla*_ACT-24_ (C) *bla*_TEM-1_ (A) *bla*_KPC-4_ (A, CP) *bla*_OXA-1_ (D)
*E. hormaechei*	78	2024	IP	Respiratory tract	≥64 (R)	≥64 (R)	≤0.25 (S)	*bla*_ACT-24_ (C) *bla*_CTX-M-15_ (A, ES) *bla*_TEM-1_ (A) *bla*_OXA-1_ (D)
*E. hormaechei*	93	2024	OP	Urine	≥64 (R)	≥64 (R)	≥16 (R)	*bla*_ACT-17_ (C) *bla*_CTX-M-15_ (A, ES) *bla*_TEM-1_ (A) *bla*_NDM-7_ (B, CP) *bla*_OXA-1_ (D)
*E. hormaechei*	109	2024	IP	Blood	≥64 (R)	≥64 (R)	≤0.25 (S)	*bla*_ACT_ (C)
*E. hormaechei*	113	2022	OP	Biliary	≥64 (R)	≥64 (R)	0.5 (S)	*bla*_ACT-56_ (C)
*E. hormaechei*	114	2022	ED	Urine	≥64 (R)	≥64 (R)	≥16 (R)	*bla*_ACT-16_ (C) *bla*_TEM-1_ (A) *bla*_NDM-5_ (B, CP)
*E. hormaechei*	124	2023	IP	Urine	≥64 (R)	≥64 (R)	≥16 (R)	*bla*_ACT-17_ (C) *bla*_SHV-12_ (A, ES) *bla*_KPC-3_ (A, CP) *bla*_OXA-1_ (D)
*E. hormaechei*	151	2022	IP	Respiratory tract	≥64 (R)	≥64 (R)	4 (R)	*bla*_ACT-17_ (C) *bla*_KPC-4_ (A, CP) *bla*_TEM_ (A) *bla*_OXA-1_ (D)
*E. hormaechei*	171	2022	ED	Urine	≥64 (R)	32 (R)	≤0.25 (S)	*bla*_ACT-45_ (C) *bla*_CTX-M-15_ (A, ES) *bla*_TEM-1_ (A) *bla*_OXA-1_ (D)
*E. hormaechei*	269	2022	IP	Respiratory tract	≥64 (R)	≥64 (R)	1 (S)	*bla*_ACT-37_ (C) *bla*_CTX-M-15_ (A, ES) *bla*_TEM-1_ (A) *bla*_NDM-1_ (B, CP) *bla*_OXA-1_ (D)
*E. hormaechei*	428/528	2023	IP	Respiratory tract	≥64 (R)	≥64 (R)	≥16 (R)	*bla*_ACT_ (C) *bla*_CTX-M-15_ (A, ES) *bla*_TEM-1_ (A) *bla*_NDM-1_ (B, CP) *bla*_OXA-1_ (D)
*E. hormaechei*	1,472	2022	ED	Urine	≥64 (R)	32 (R)	1 (S)	*bla*_ACT-15_ (C)
*E. hormaechei*	1,707	2024	IP	Respiratory tract	≥64 (R)	≥64 (R)	≤0.25 (S)	*bla*_ACT-41_ (C) *bla*_CTX-M-55_ (A, ES)
*E. kobei*	56	2023	IP	Rectal	≥64 (R)	≥64 (R)	0.5 (S)	*bla*_ACT-15_ (C)
*K. aerogenes*	4	2022	IP	Respiratory tract	≥64 (R)	≥64 (R)	≤0.25 (S)	Unspecified *ampC* (C) *bla*_CTX-M-15_ (A, ES) *bla*_TEM-1_ (A)
*K. aerogenes*	93	2022	IP	Intraabdominal	≥64 (R)	≥64 (R)	2 (I)	Unspecified *ampC* (C)
*K. aerogenes*	93	2021	IP	Rectal	≥64 (R)	≥64 (R)	8 (R)	Unspecified *ampC* (C)
*K. aerogenes*	93	2023	IP	Respiratory tract	≥64 (R)	≥64 (R)	4 (R)	Unspecified *ampC* (C)
*K. aerogenes*	128	2023	IP	Urine	≥64 (R)	32 (R)	≤0.25 (S)	Unspecified *ampC* (C)
*K. aerogenes*	304	2021	OP	Urine	≥64 (R)	≥64 (R)	≤0.25 (S)	Unspecified *ampC* (C) *bla*_CTX-M-15_ (A, ES)
*K. aerogenes*	363	2022	IP	Skin/tissue	≥64 (R)	≥64 (R)	8 (R)	Unspecified *ampC* (C)

^*a*^ ST, sequence type. Setting, clinical setting in which specimen was collected. IP, inpatient. OP, outpatient. ED, emergency department. CRO, ceftriaxone. FEP, cefepime. MEM, meropenem. MIC, minimum inhibitory concentration. MICs are in units of µg/mL. If disk diffusion was used for testing, the zone diameter is listed instead with units indicated. The interpretive category according to the breakpoints in the 35^th^ edition of the CLSI M100 are indicated in parenthesis after each value. S, susceptible. I, intermediate. R, resistant. Beta-lactamase genes detected via annotation of whole-genome sequences are listed with the Ambler class (A–D) in parenthesis. Presumed extended-spectrum beta-lactamase genes are annotated as “ES,” presumed carbapenemase genes are annotated as “CP,” and plasmid-borne *ampC *beta-lactamase genes are annotated as “PAC”.

**Fig 4:**
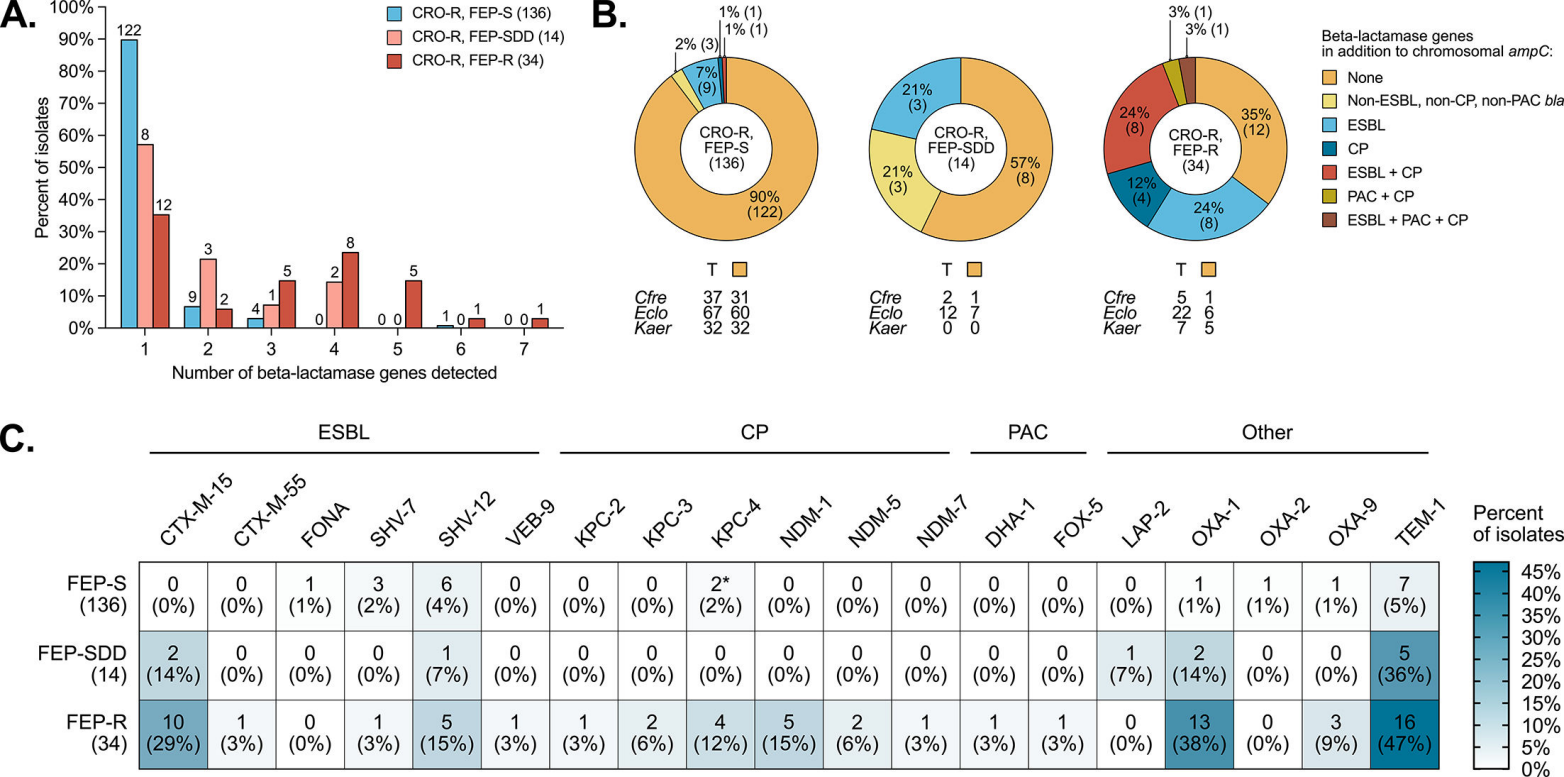
Beta-lactamase gene carriage of *Cfre*, *Eclo*, and *Kaer* clinical isolates. (**A**) Number of beta-lactamase genes detected in ceftriaxone (CRO)-resistant (R) isolates (all three organism groups pooled) stratified by the cefepime (FEP) susceptibility category. S, susceptible. SDD, susceptible dose-dependent. The number above each bar indicates the absolute number of isolates represented. The total number of isolates in each category is in parenthesis in the legend. For a breakdown by the organism group, refer to Fig. S2A and B. (**B**) Types of beta-lactamases detected in CRO-R, FEP-S; CRO-R, FEP-SDD; and CRO-R, FEP-R isolates. ESBL, extended-spectrum beta-lactamase. CP, carbapenemase. PAC, plasmid-borne *ampC*. The total number of isolates is indicated in parenthesis in the center of each circle. Each section is labeled with the percentage of isolates with the absolute number of isolates in parenthesis. The total number of isolates (T) and the number of isolates encoding chromosomal *ampC* only (orange square) from each organism group are indicated below each circle. The “non-ESBL, non-CP, non-PAC” category refers to isolates that have only non-ESBL, non-CP, non-PAC beta-lactamase genes in addition to chromosomal *ampC*; some isolates in the other categories also have non-ESBL, non-CP, non-PAC beta-lactamase genes. Refer to Tables 2 and 3 and Table S3 for a full list of beta-lactamase genes carried by each isolate. (**C**) Beta-lactamase genes present in CRO-R, FEP-S; CRO-R, FEP-SDD; and CRO-R, FEP-R isolates. Each square is labeled with the number of isolates bearing that beta-lactamase gene with the percentage in parenthesis. *, an intact *bla*_KPC-4_ gene was detected in two FEP-S isolates. Because KPC-4 (a carbapenemase) should confer cefepime resistance, it is possible that the plasmid bearing *bla*_KPC-4_ was lost when these isolates were sub-cultured for susceptibility testing.

Increasing FEP MICs were generally associated with carriage of increasing numbers of additional beta-lactamase genes: 43% (6/14) of FEP-SDD and 65% (22/34) of FEP-R isolates had additional beta-lactamase genes compared to 10% (14/136) of FEP-S isolates (Fig. 4A and B; Fig. S2A and B). These encoded ESBLs, carbapenemases, plasmid-borne AmpCs, and other Ambler class A and D beta-lactamases (Fig. 4B and C). *bla*_CTX-M_ was the most common ESBL, carried by 32% (11/34) of FEP-R, 14% (2/14) of FEP-SDD, and 0% (0/136) of FEP-S isolates (Fig. 4C). Other ESBLs, including *bla*_FONA_, *bla*_SHV-7_, *bla*_SHV-12_, and *bla*_VEB-9_, were present in 21% (7/34) of FEP-R, 7% (1/14) of FEP-SDD, and 7% (10/136) of FEP-S isolates. The carbapenemase genes *bla*_KPC_ and *bla*_NDM_ were common in FEP-R isolates, among which 21% (7/34) had a *bla*_KPC_ and 24% (8/34) had a *bla*_NDM_, and virtually absent from FEP-SDD and FEP-S isolates. In addition, 2 FEP-R isolates had plasmid-borne *ampC* genes, and a high proportion of FEP-R isolates (38%, 13/34) carried *bla*_OXA-1_. Overall, 35% (12/34) of FEP-R isolates carried only chromosomal *ampC* (Fig. 4B). The proportion was greatest for *Kaer* (71%, 5/7 vs 20%, 1/5 for *Cfre,* and 27%, 6/22 for *Eclo*).

## DISCUSSION

AmpC-producing organisms represent a unique subset of Enterobacterales for which prior research has shown that FEP can safely be used to treat isolates that exhibit, or will exhibit, CRO resistance (1). For isolates that are FEP-R, or FEP-SDD with an ESBL present, carbapenems are recommended (1). Rapid molecular diagnostics that identify AmpC-producing organisms along with important resistance determinants, including the ESBL *bla*_CTX-M_, are increasingly used to guide the selection of FEP vs a carbapenem, with FEP often recommended if no resistance determinants are detected. While this approach is undoubtedly helpful in most cases, its safety depends on the proportion of FEP-R and FEP-SDD isolates (or, for infections typically treated with high-dose cefepime, ESBL-bearing FEP-SDD isolates) that possess a resistance determinant represented on the molecular panel.

Here, using microbiologic data from over 19,000 AmpC producers, we show that the overall prevalence of FEP-SDD and -R is 3.2% (614/19,366), with substantial variability by organism, patient setting, and body site. Furthermore, we show that FEP-SDD and -R isolates exhibit diverse beta-lactamase resistance determinants, ranging from the carriage of chromosomal *ampC* only to carriage of multiple beta-lactamase genes that include ESBL genes, carbapenemase genes, and plasmid-borne *ampC*. Overall, these data support empiric use of cefepime for AmpC producers, especially *Cfre* and *Kaer*, for which FEP-SDD/R rates were less than 2% and 1%, respectively. However, the variability in FEP susceptibility rates suggests caution is warranted in respiratory infections with *Cfre* and *Eclo* and in inpatients with *Eclo* infections, especially bloodstream infections. The prevalence of FEP resistance in *Eclo* observed here was lower than in reports from Europe and Taiwan (10%–36%) (4–6), underscoring the importance of local characterization.

Our analysis of molecular mechanisms underlying FEP-SDD and FEP-R phenotypes found that 4.3% (5/117) of *Eclo* and *Kaer* bloodstream isolates detected on the BCID2 panel lacked a beta-lactamase resistance gene target, yet ultimately exhibited a FEP-SDD (3/117) or FEP-R (2/117) phenotype. Depending on the prevalence of non-*bla*_CTX-M_ ESBL carriage among FEP-SDD bloodstream isolates, which we were not able to fully evaluate here, this suggests that up to 1 in 25 patients with these BCID2 results could receive inadequate or inappropriate empiric therapy with high-dose cefepime, which is typically used over standard-dose cefepime to treat sepsis. Further, our WGS analysis found that 85.7% (12/14) of FEP-SDD isolates and 38.2% (13/34) of FEP-R isolates lacked a multiplex PCR panel beta-lactamase target (Fig. S2C), showing that absence of a beta-lactamase target does not rule out a FEP-SDD or FEP-R phenotype. This highlights the need for individualized risk assessment based on prior microbiological information and healthcare/antibiotic exposure to help guide result interpretation and treatment recommendations.

In addition, 21.4% (3/14) of sequenced FEP-SDD isolates carried an ESBL (two *bla*_CTX-M-15_ and one *bla*_SHV-12_). While much lower than the 89% ESBL carriage rate reported in a Taiwanese study (4), this finding suggests substantial ESBL carriage among FEP-SDD isolates in our region. To guide clinicians in choosing between high-dose FEP and a carbapenem, laboratories with the requisite resources could consider making ESBL testing for isolates that test FEP-SDD available. Phenotypic ESBL testing is challenging in AmpC producers due to interference from AmpC expression, but a modified version of the standard Clinical Laboratory Standards Institute (CLSI) phenotypic ESBL confirmatory test (7) that uses antibiotic disks containing boronic acid, an inhibitor of AmpC activity, has been described (8). An FDA-cleared lateral flow assay for the detection of CTX-M in cultured isolates is also available (9) and could be a useful tool in areas where CTX-M is the dominant ESBL among AmpC producers.

Our results highlight variability in FEP resistance profiles and underlying genetic characteristics among *Cfre*, *Eclo*, and *Kaer*. Similar to European studies (5, 6), we found that *Eclo* had the highest overall prevalence of FEP-SDD and FEP-R phenotypes. However, *Cfre* harbored the highest proportion of CRE, with about 25% of FEP-SDD/R isolates displaying resistance to MEM vs 10% for *Eclo* and *Kaer. Kaer* had the lowest prevalence of FEP-SDD/R phenotypes and tended to encode fewer beta-lactamases than *Cfre* and *Eclo*. Most *Kaer* FEP-R isolates—71%—encoded only *ampC*, and three were also MEM-R. This suggests that FEP, and potentially carbapenem, resistance is more often conferred by mechanisms such as *ampC* derepression and/or porin mutations in *Kaer* than by acquisition of ESBLs and carbapenemases, echoing previous reports (10, 11).

In terms of limitations, we inferred some susceptibility results and applied only CLSI breakpoints; use of the European Committee on Antimicrobial Susceptibility Testing (EUCAST) breakpoints (Table S3) (12) likely would have yielded higher rates of FEP resistance. Our study was regional in nature, and thus the applicability of our findings to other geographic areas is unknown. Due to the small sample size and use of a convenience sample of CRO-R isolates, our WGS data might not reflect general patterns of beta-lactamase gene carriage among AmpC producers. The investigation of resistance determinants involving *ampC* regulatory pathways and non-enzymatic factors was outside the scope of our study, but future work to determine their influence on FEP susceptibility could be informative. Finally, analysis of patient outcomes was outside the scope of our study. Future studies that stratify outcomes by the FEP susceptibility category and resistance determinants would be valuable to guide recommendations on molecular diagnostics and empiric treatment strategies.

Overall, our results highlight diversity in phenotypic susceptibility to FEP among AmpC producers across clinical settings and in underlying molecular mechanisms, suggesting the need for careful consideration of patient characteristics when using rapid molecular diagnostics to guide clinical decision-making. A thorough understanding of FEP resistance mechanisms, their local prevalence, and their influence on patient outcomes is necessary to ensure optimal antibiotic treatment for each patient.

## MATERIALS AND METHODS

### Study design and setting

We conducted a retrospective, descriptive study of patients who received care between May 2015 and June 2024 at any facility within the Mass General Brigham (MGB) health system, which encompasses two quaternary care hospitals, seven community hospitals, three specialty hospitals, and several outpatient centers in Massachusetts and New Hampshire, and at the Dana Farber Cancer Institute.

### Data procurement

We queried the MGB clinical data warehouse for all specimens that grew *Cfre*, *Eclo*, or *Kaer*. All isolates were obtained as part of routine diagnostic workups and identified to the species or complex level per laboratory protocol. Phenotypic antimicrobial susceptibility testing (AST) was performed on each isolate as part of routine clinical care using automated broth microdilution (VITEK 2 from bioMérieux, Marcy-l'Étoile, France, or Sensititre from Thermo Fisher Scientific, Waltham, MA, USA), gradient diffusion (ETEST from bioMérieux, Marcy-l'Étoile, France), or disk diffusion (various commercial discs) depending on the AST protocol used by the site where the isolate was obtained.

### Isolate selection and AST interpretation

We included one isolate of each organism per patient, retaining the earliest isolate that was tested for susceptibility to both CRO and FEP for patients with multiple isolates (Table S4). We determined interpretive susceptibility categories for CRO and FEP using the MIC and zone diameter breakpoints for Enterobacterales listed in Table 2A-1 of the 35th edition of the CLSI M100 Performance Standards for Antimicrobial Susceptibility Testing (Table 1) (7). For 21.0% of isolates (4,232/20,130), AST results were not interpretable (NI) or not available (NA). Most of these (2,970/20,130, 14.8%) were CRO-S or CRO-I with FEP results NI or NA; we inferred a FEP-S result for these isolates. For the remaining 1,262 isolates, we discarded those for which susceptibility to CRO or FEP was not tested (764/20,130, 3.8%) and classified the rest (498/20,130, 2.5%) according to the interpretive categories reported in the laboratory information system at the time the isolate was tested. See the supplemental methods for additional details.

### WGS analysis

We reviewed WGS data from 184 CRO-R isolates (136 FEP-S, 14 FEP-SDD, and 34 FEP-R) obtained from diverse body sites that were sequenced in the Brigham and Women’s Hospital clinical microbiology laboratory between 2021 and 2024. DNA was extracted using the EZ1 Advanced XL instrument (Qiagen, Valencia, CA). Libraries prepared using the Illumina DNA Prep Kit were sequenced on the MiSeq (Illumina, Inc., San Diego, CA). Quality control was performed on raw reads using Trimmomatic v0.39 (13) and FastQC v0.11.9 (14). SPAdes v3.15.3 (15) was used to generate draft assemblies, with quality assessed by QUAST v5.0.3 (16). Kraken v2.1.2 (01/31/2023) and StrainSeeker v1.5 (17) were used to assign genus and species. Multilocus sequence typing (MLST) was performed using an open-source package (https://github.com/tseemann/mlst). Antimicrobial resistance (AMR) genes were annotated using AMRFinderPlus (version 4.0.3, database version 2024-12-18.1) (18, 19).

### BIOFIRE BCID2 analysis

Three hospitals in the MGB network ran the BIOFIRE BCID2 panel (bioMérieux, Salt Lake City, UT, USA) on positive blood culture bottles at the time of our study (implemented between 2020 and 2024). The assay was run on one bottle per patient per week and only repeated within that period if the Gram stain of a newly positive bottle differed from an earlier Gram stain. We identified all blood cultures in which *Eclo* or *Kaer* targets were detected on the BCID2. Since *Cfre* is not an individual target on the BCID2, we also identified blood cultures in which the Enterobacterales order-level target was detected without a complex- or species-level target that later grew *Cfre*. We then collected results of AST performed on the subsequently cultured organisms, excluding bottles for which the detected organism did not grow. For isolates not tested for FEP, we inferred a susceptible result for CRO-susceptible or CRO-intermediate isolates and excluded CRO-resistant isolates.

### Statistical analysis

Proportions were compared using the chi-square test (performed in Python with the statsmodels.stats.proportion.proportions_chisquare function), with *P*-values adjusted for multiple comparisons using the Bonferroni correction.

## SUPPLEMENTAL MATERIAL

Supplemental material aac.01661-25-s0001.docx Supplemental methods; Fig. S1 and S2; Tables S1 and S3.

Table S2 aac.01661-25-s0002.xlsx Beta-lactamase genes detected in sequenced ceftriaxone-resistant, cefepime-susceptible isolates.

Table S4 aac.01661-25-s0003.xlsx Number of isolates included from each collection site.

ASM does not own the copyrights to Supplemental Material that may be linked to, or accessed through, an article. The authors have granted ASM a non-exclusive, world-wide license to publish the Supplemental Material files. Please contact the corresponding author directly for reuse.
