# Supplementary material for: Prevalence and genetics of cefepime resistance among the AmpC-producing organisms Citrobacter freundii complex, Enterobacter cloacae complex, and Klebsiella aerogenes
Source: Antimicrob Agents Chemother. 2026 Mar 27;70(5):e01661-25. doi: 10.1128/aac.01661-25 (PMC13148017; doi:10.1128/aac.01661-25)
Supplement: Supplemental material — Supplemental methods; Fig. S1 and S2; Tables S1 and S3. [file aac.01661-25-s0001.docx]

**SUPPLEMENTAL MATERIALS**

**Article title**

Prevalence and genetics of cefepime resistance among the AmpC-producing organisms *Citrobacter freundii* complex, *Enterobacter cloacae* complex, and *Klebsiella aerogenes*

**Authors**

Sarah M. Schrader, Zachary Pearson, Samantha Taffner, Sanjat Kanjilal, Nicole D. Pecora

**Contents**

1. Supplemental methods………………………….................................................pg 2
2. Figure S1……………………………………………………………………………..pg 3
3. Figure S2……………………………………………………………………………..pg 4
4. Table S1………………………………………………………………………………pg 5
5. Table S2 legend………………………………………………………………….. …pg 6
6. Table S3………………………………………………………………………………pg 7
7. Table S4 legend……………………………………………………………………...pg 8

**Other supplemental materials**

1. Table S2 – separate Excel file
2. Table S4 – separate Excel file

**SUPPLEMENTAL METHODS**

**Classification of isolates with ceftriaxone (CRO) and/or cefepime (FEP) susceptibility results that were not interpretable or not available**

For some isolates, the minimum inhibitory concentration (MIC) value reported (≤ 2 µg/mL or ≤ 8 µg/mL for CRO and ≤ 4 µg/mL or ≤ 8 µg/mL for FEP) did not permit distinction between the susceptible category and intermediate category for CRO or between the susceptible category and susceptible dose-dependent category for FEP and was thus not interpretable (NI). For other isolates, a numerical result was not available (NA), either because results of AST were reported in the laboratory information system (LIS) with only a categorical interpretation or because the isolate was not tested for susceptibility to CRO and/or FEP. Isolates from sites for which all isolates had NI or NA results for both CRO and FEP were excluded. Remaining isolates with NI and/or NA results were classified as follows:

- Isolates with CRO results interpreted as susceptible (CRO-S) or intermediate (CRO-I) and with NI or NA FEP (FEP-NI/NA) results (2,970 isolates): classified as FEP-S based on a lack of CRO-S isolates categorized as FEP-SDD or FEP-R among the isolates with interpretable AST results for both antibiotics.
- CRO-R, FEP-NI/NA isolates (631 isolates): isolates not tested for susceptibility to FEP were excluded. Remaining isolates were classified according to the FEP interpretive category reported in the LIS.
- CRO-NI/NA, FEP-S isolates (214 isolates): isolates not tested for susceptibility to CRO were excluded. Remaining isolates were classified according to the CRO interpretive category reported in the LIS.
- CRO-NI/NA, FEP-SDD or FEP-R (5 isolates): classified as CRO-R based on a lack of FEP-SDD or FEP-R isolates classified as CRO-S or CRO-I among the isolates with interpretable AST results for both antibiotics.
- CRO-NI, FEP-NI isolates (225 isolates): classified according to the CRO and FEP susceptibility categories reported in the LIS.
- CRO-NA, FEP-NA isolates (187 isolates): isolates not tested for susceptibility to CRO and/or FEP were excluded. Remaining isolates were classified according to the CRO and FEP interpretive categories reported in the LIS.

A total of 3,468 isolates (17.2% of 20,130 total isolates) were reclassified and 764 (3.8% of 20,130) were excluded based on the above criteria.

**SUPPLEMENTAL FIGURES AND TABLES**

**Figure S1.** *bla*_ACT_ alleles detected in *E. cloacae* (*Eclo*) complex isolates stratified by cefepime (FEP) susceptibility category. The number above each bar indicates the absolute number of isolates represented. The total number of isolates in each category is in parenthesis in the legend. CRO, ceftriaxone. S, susceptible. SDD, susceptible dose-dependent. R, resistant.

**
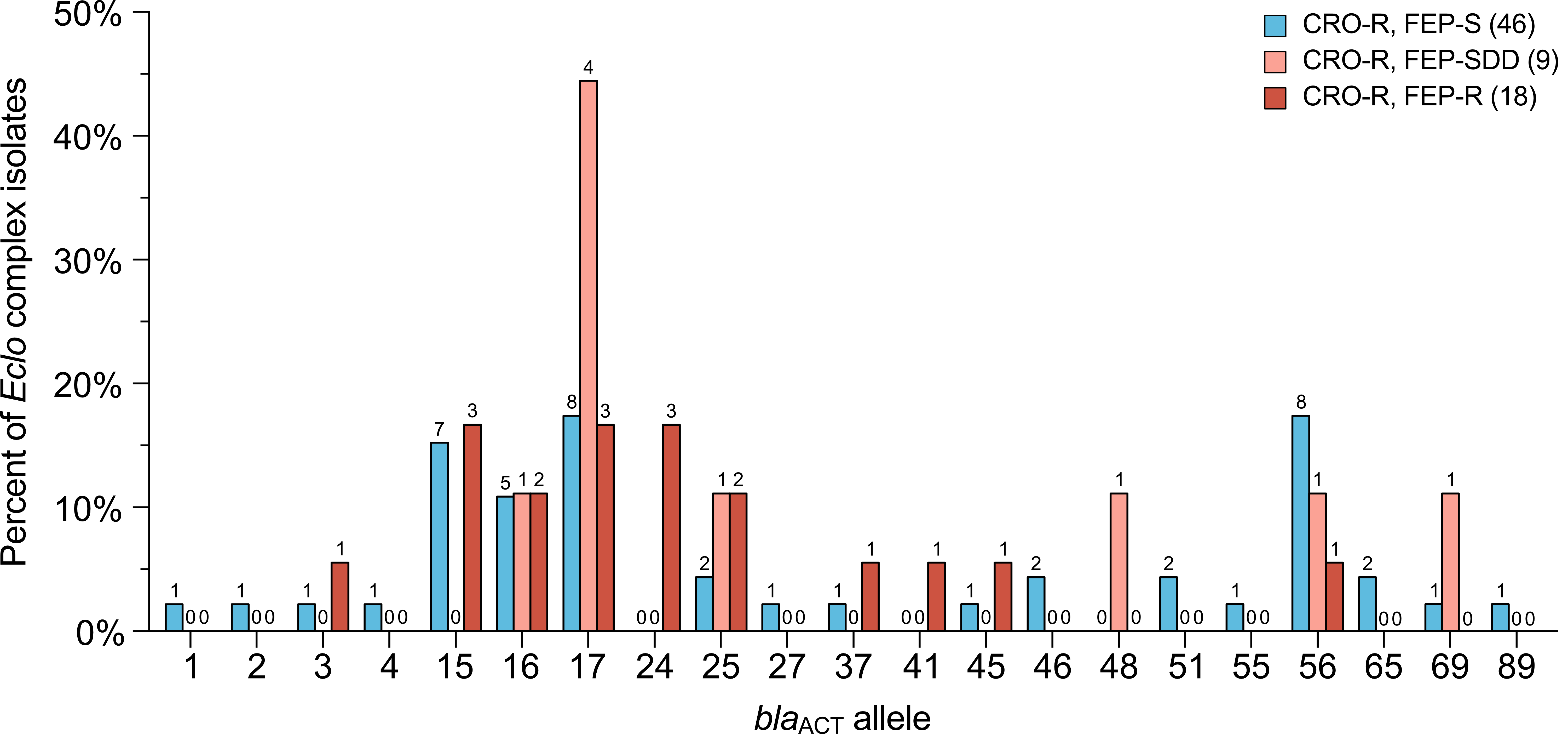
**

**Figure S2.** A-B) Number of beta-lactamase genes detected in A) ceftriaxone-resistant, cefepime-susceptible (FEP-S) isolates and B) ceftriaxone-resistant, cefepime-susceptible dose-dependent and -resistant (pooled; FEP-SDD/R) isolates by organism group. *Cfre*, *C. freundii* complex. *Eclo*, *E. cloacae* complex. *Kaer*, *K. aerogenes*. The number above each bar indicates the absolute number of isolates represented. The total number of isolates in each category is in parenthesis in the legend. C) Presence of a multiplex PCR panel beta-lactamase (*bla*) target gene among sequenced FEP-SDD and FEP-R isolates. The total number of isolates (all three organism groups pooled) is indicated in parenthesis in the center of each circle. Sections are labeled with the percentages they represent and the absolute number of isolates in parenthesis. The total number of isolates (T) and the number of isolates with or without a PCR panel target for each organism is indicated below each circle.


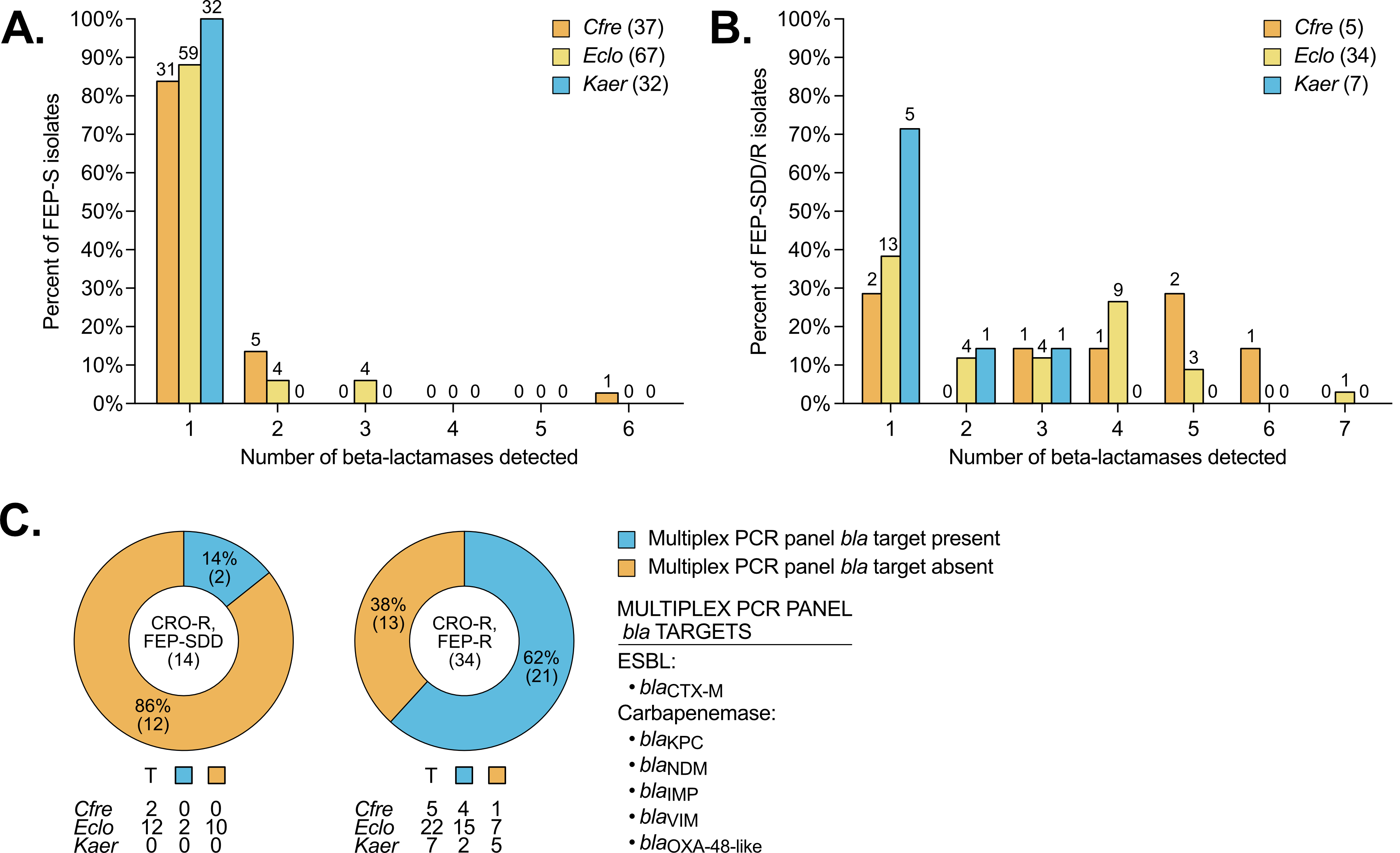


**Table S1. Ceftriaxone (CRO) and cefepime (FEP) susceptibility profiles among *C. freundii* complex (*Cfre*), *E. cloacae* complex (*Eclo*), and *K. aerogenes* (*Kaer*) isolates.** S, susceptible. SDD, susceptible dose-dependent. I, intermediate. R, resistant.

|  | *Cfre* | | *Eclo* | | *Kaer* | |
| --- | --- | --- | --- | --- | --- | --- |
|  | NUMBER | PERCENT | NUMBER | PERCENT | NUMBER | PERCENT |
| CRO-S_FEP-S | 3781 | 80.67% | 7696 | 77.90% | 4139 | 86.23% |
| CRO-S_FEP-SDD | 0 | 0.00% | 1 | 0.01% | 3 | 0.06% |
| CRO-S_FEP-R | 0 | 0.00% | 0 | 0.00% | 1 | 0.02% |
| CRO-I_FEP-S | 36 | 0.77% | 146 | 1.48% | 26 | 0.54% |
| CRO-I_FEP-SDD | 0 | 0.00% | 0 | 0.00% | 0 | 0.00% |
| CRO-I_FEP-R | 0 | 0.00% | 0 | 0.00% | 0 | 0.00% |
| CRO-R_FEP-S | 793 | 16.92% | 1543 | 15.62% | 587 | 12.23% |
| CRO-R_FEP-SDD | 18 | 0.38% | 194 | 1.96% | 16 | 0.33% |
| CRO-R_FEP-R | 59 | 1.26% | 299 | 3.03% | 28 | 0.58% |
| TOTAL INCLUDED | 4687 | 100.00% | 9879 | 100.00% | 4800 | 100.00% |

**Table S2. Beta-lactamase genes detected in sequenced ceftriaxone-resistant, cefepime-susceptible isolates.** ST, sequence type. Setting, clinical setting in which specimen was collected. IP, inpatient. OP, outpatient. ED, emergency department. CRO, ceftriaxone. FEP, cefepime. MEM, meropenem. MIC, minimum inhibitory concentration. MICs are in units of µg/mL. The interpretive category according to the breakpoints in the 35^th^ edition of the CLSI M100 are indicated in parenthesis after each value. S, susceptible. SDD, susceptible dose-dependent. I, intermediate. R, resistant. Beta-lactamase genes detected via annotation of whole-genome sequences are listed with the Ambler class (A-D) in parenthesis. Presumed extended-spectrum beta-lactamases are annotated as “ES”, and presumed carbapenemases are annotated as “CP”. Note that an intact *bla*_KPC-4_ gene was detected in two isolates. Because KPC-4 (a carbapenemase) should confer cefepime resistance, it is possible that the plasmid bearing *bla*_KPC-4_ was lost when the isolates were sub-cultured for susceptibility testing.

(See separate Excel file)

**Table S3. European Committee on Antimicrobial Susceptibility Testing (EUCAST) breakpoints for Ceftriaxone (CRO), cefepime (FEP), and meropenem (MEM).** From EUCAST Clinical Breakpoints version 16.0 (2026). S, susceptible, standard dosing regimen. I, susceptible, increased exposure. R, resistant. MIC, minimum inhibitory concentration.

|  | CRO | | FEP | | MEM | |
| --- | --- | --- | --- | --- | --- | --- |
| Interpretive category | MIC (µg/mL) | Zone diameter (mm) | MIC (µg/mL) | Zone diameter (mm) | MIC (µg/mL) | Zone diameter (mm) |
| S | ≤ 1 | ≥ 27 | ≤ 1 | ≥ 27 | ≤ 2 | ≥ 22 |
| I | 2 | 24-26 | 2-4 | 24-26 | 4-8 | 17-21 |
| R | ≥ 4 | ≤ 23 | ≥ 8 | ≤ 23 | ≥ 16 | ≤ 16 |

**Table S4. Number of isolates included from each collection site.**

(See separate Excel file)
